# Supplementary material for: An experimental test of the influence of microbial manipulation on sugar kelp (Saccharina latissima) supports the core influences host function hypothesis
Source: Appl Environ Microbiol. 2025 May 29;91(6):e00301-25. doi: 10.1128/aem.00301-25 (PMC12175540; doi:10.1128/aem.00301-25)
Supplement: Supplemental figures — Figures S1 to S4. [file aem.00301-25-s0001.docx]

**An experimental test of the influence of microbial manipulation on sugar kelp (*Saccharina latissima*) supports the the core influences host function hypothesis**

Supplemental Figures:


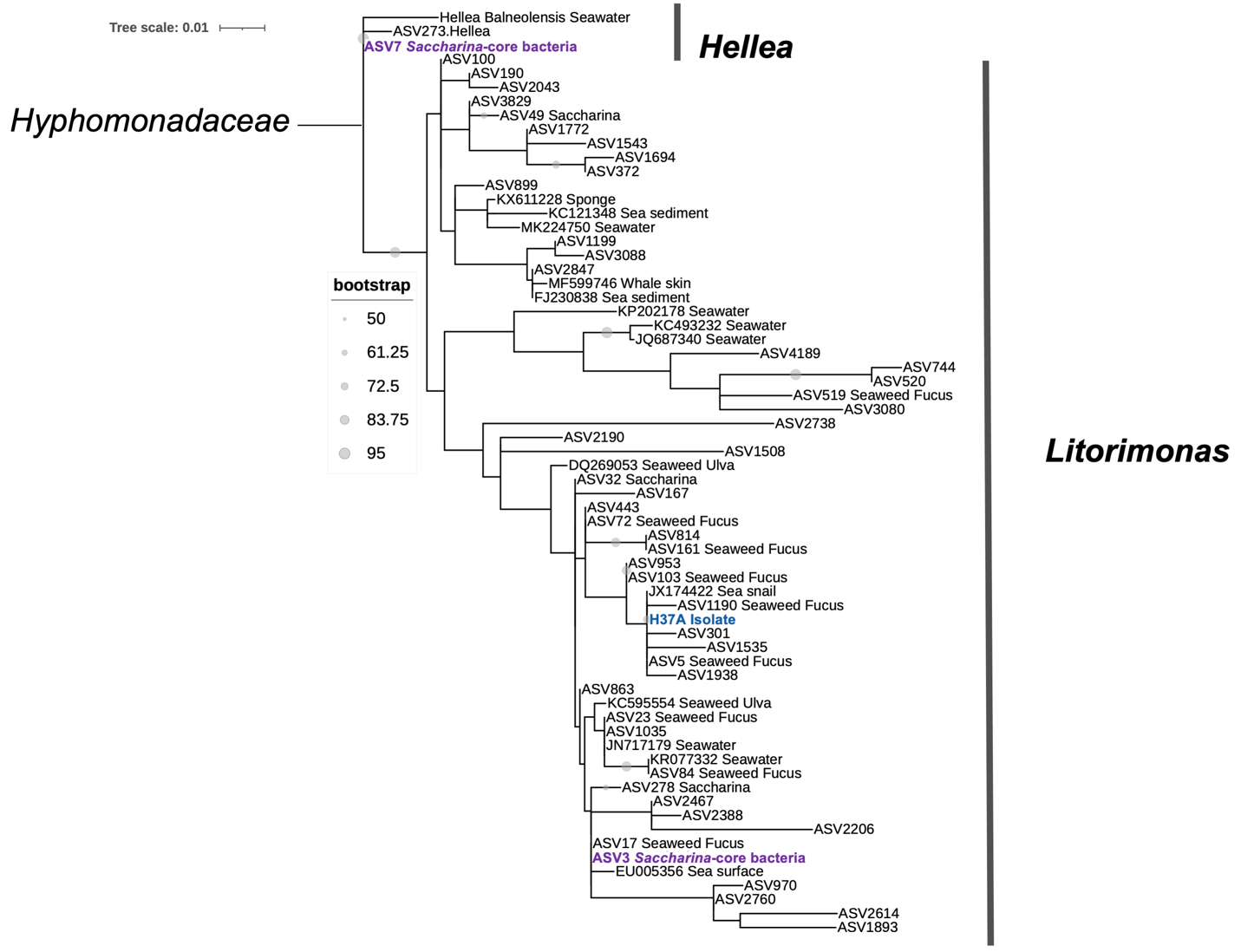


**FIG S1 | A phylogenetic tree of *Hyphomonadaceae* clade bacterial sequences.** The tree was constructed using RAxML and rapid bootstrapping. The core ASVs of *S. latissima*, identified in this study are colored purple, while the sequences of the cultured bacterial isolates in the laboratory are colored blue. Other sequences obtained from GenBank are annotated with information on the isolate source. Only bacterial sequences within branches with bootstrap values greater than 50 are shown.


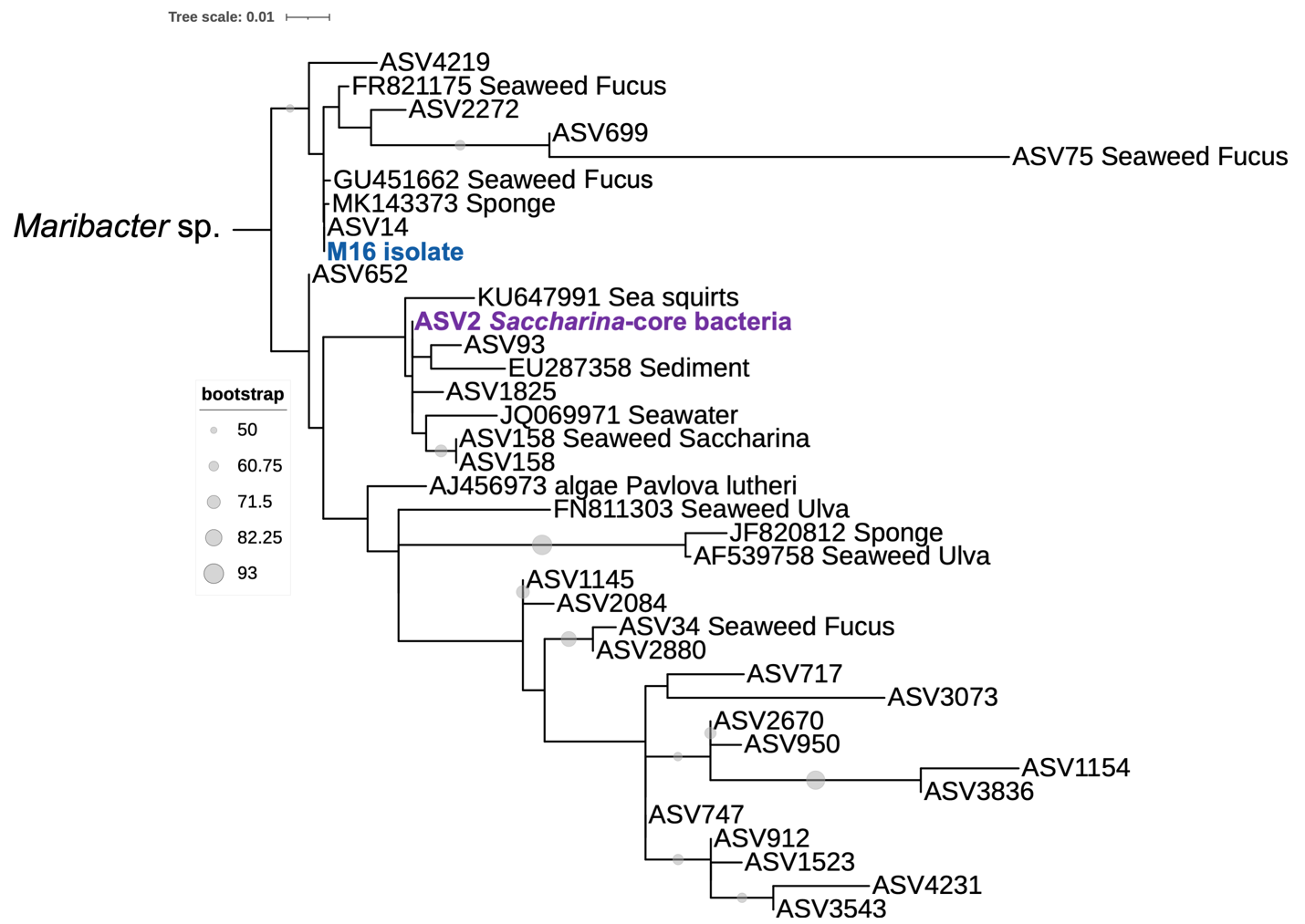


**FIG S2 | A phylogenetic tree of *Maribacter* clade bacterial sequences.** Other notes as in Fig. S1.

**
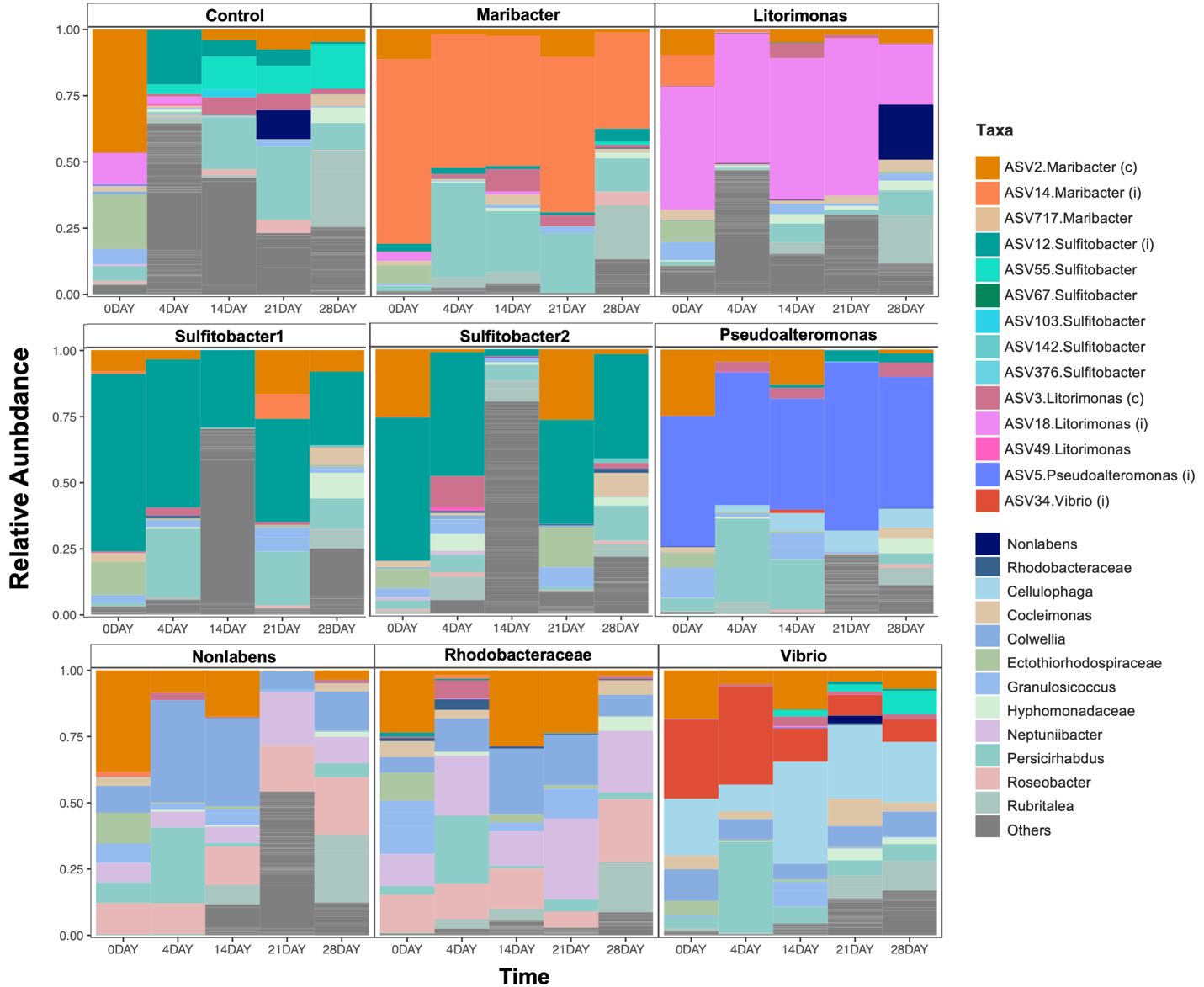
 FIG S3 | Taxonomic bar plot of bacterial community in co-culture trial 4 over the 28-days. Most abundant genera across all trials plus Data from amplicon sequencing.** The bacterial isolated that were inoculated are indicated (i) used for microbial manipulation are displayed at the ASV level to differentiate between different species within the core genera. The core ASVs (c) identified by IndVal analysis that were associated with wild S. latissima were also present in this laboratory-based microcosm. A water change occurred prior to the 14-day sampling.


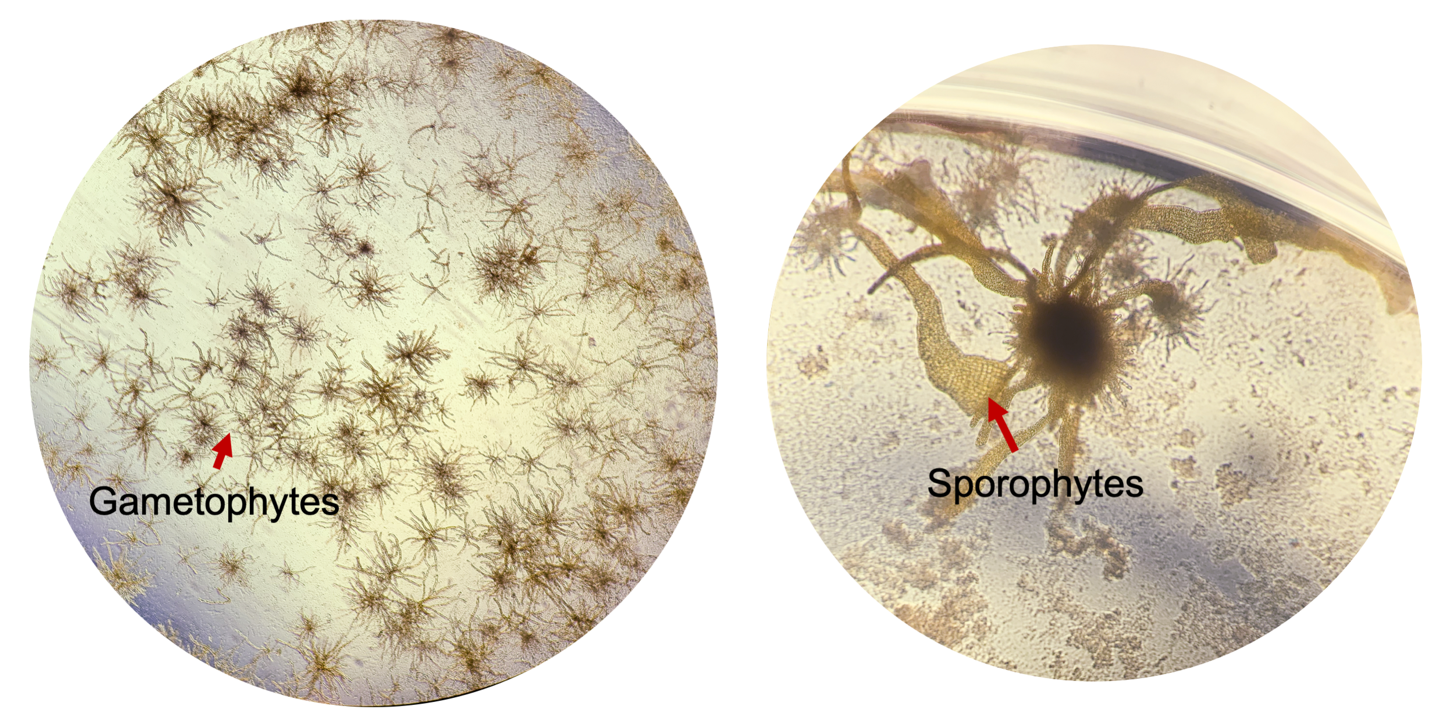


**FIG S4 | Morphological characteristics of *S. latissima* between gametophytes and sporophytes under microscopy.**

Supplemental Table Captions:

**Table S1| List of cultured isolates, their taxonomy and IAA production.**

**Table S2| Indicator species (IndVal) analysis for wild *S. latissima* against environments at bacterial ASV level.**

**Table S3| Overall frequency and abundance of bacterial genus in the wild *Saccharia latissima*-microbiome.**

**Table S4| Co-culture trial results.**
